# A Delta-radiomics model for preoperative evaluation of Neoadjuvant chemotherapy response in high-grade osteosarcoma

**DOI:** 10.1186/s40644-019-0283-8

**Published:** 2020-01-14

**Authors:** Peng Lin, Peng-Fei Yang, Shi Chen, You-You Shao, Lei Xu, Yan Wu, Wangsiyuan Teng, Xing-Zhi Zhou, Bing-Hao Li, Chen Luo, Lei-Ming Xu, Mi Huang, Tian-Ye Niu, Zhao-Ming Ye

**Affiliations:** 1 Musculoskeletal Tumor Center, Department of Orthopaedics, The Second Affiliated Hospital, Zhejiang University School of Medicine, Zhejiang, 310009 Hangzhou China; 2 Institute of Orthopaedics Research, No.88 Jiefang Road, Hangzhou City, Zhejiang Province, 310009 China; 3 Sir Run Run Shaw Hospital, Zhejiang University School of Medicine, Institute of Translational Medicine, Zhejiang University, Zhejiang, Hangzhou China; 4 College of Biomedical Engineering &Instrument Science, Zhejiang University, Zhejiang, Hangzhou China; 5 Department of Orthopaedics, Ninghai First Hospital, Ningbo, Zhejiang, 315600 China; 6 Department of Pediatrics, Children’s Hospital, Zhejiang University School of Medicine, Zhejiang, 310052 Hangzhou China; 7 Department of Radiology, The Second Affiliated Hospital, Zhejiang University School of Medicine, Zhejiang, 310009 Hangzhou China; 8 Department of Radiation Oncology, Duke University Medical Center, Durham, North Carolina, 27708 USA; 9 Nuclear & Radiological Engineering and Medical Physics Programs, Woodruff School of Mechanical Engineering, Georgia Institute of Technology, 770 State Street, Boggs 385, Atlanta, GA 30332-0745 USA

**Keywords:** High-grade osteosarcoma, Chemotherapy response evaluation, CT, Delta-radiomics, Machine learning

## Abstract

**Background:**

The difficulty of assessment of neoadjuvant chemotherapeutic response preoperatively may hinder personalized-medicine strategies that depend on the results from pathological examination.

**Methods:**

A total of 191 patients with high-grade osteosarcoma (HOS) were enrolled retrospectively from November 2013 to November 2017 and received neoadjuvant chemotherapy (NCT). A cutoff time of November 2016 was used to divide the training set and validation set. All patients underwent diagnostic CTs before and after chemotherapy. By quantifying the tumor regions on the CT images before and after NCT, 540 delta-radiomic features were calculated. The interclass correlation coefficients for segmentations of inter/intra-observers and feature pair-wise correlation coefficients (Pearson) were used for robust feature selection. A delta-radiomics signature was constructed using the lasso algorithm based on the training set. Radiomics signatures built from single-phase CT were constructed for comparison purpose. A radiomics nomogram was then developed from the multivariate logistic regression model by combining independent clinical factors and the delta-radiomics signature. The prediction performance was assessed using area under the ROC curve (AUC), calibration curves and decision curve analysis (DCA).

**Results:**

The delta-radiomics signature showed higher AUC than single-CT based radiomics signatures in both training and validation cohorts. The delta-radiomics signature, consisting of 8 selected features, showed significant differences between the pathologic good response (pGR) (necrosis fraction ≥90%) group and the non-pGR (necrosis fraction < 90%) group (*P* < 0.0001, in both training and validation sets). The delta-radiomics nomogram, which consisted of the delta-radiomics signature and new pulmonary metastasis during chemotherapy showed good calibration and great discrimination capacity with AUC 0.871 (95% CI, 0.804 to 0.923) in the training cohort, and 0.843 (95% CI, 0.718 to 0.927) in the validation cohort. The DCA confirmed the clinical utility of the radiomics model.

**Conclusion:**

The delta-radiomics nomogram incorporating the radiomics signature and clinical factors in this study could be used for individualized pathologic response evaluation after chemotherapy preoperatively and help tailor appropriate chemotherapy and further treatment plans.

## Background

Osteosarcoma is the most common primary malignant bone tumor in children and adolescents with an incidence rate of 2–3 per million [1], and nearly 90% cases are classified as high-grade osteosarcomas (HOS) [2]. The standard-of-care treatment is neoadjuvant chemotherapy (NCT), subsequent surgical resection and adjuvant chemotherapy [3]. With the introduction of NCT, the long-term survival rate of localized osteosarcoma patients has significantly improved and the 5-year survival rate is now estimated at approximately 60–70% [4]. However, there are still some patients whose prognoses are not ideal, especially in patients with poor histologic responses after NCT [4, 5].

Accurate identification of histologic responses to chemotherapy in patients with HOS is crucial for prognoses and treatment strategy decisions [6]. The chemotherapy strategy is adjusted according to the poor initial response to osteosarcoma during the course of treatment. Some patients with poor pathologic responses, however, are not even suitable to undergo limb salvage surgery. But the exact chemotherapeutic response assessment needs to be based on pathological findings after surgical resection [7]. Accordingly, evaluation of pathologic responses using non-invasive approaches could be important.

Previously, a patient’s pathologic response was usually estimated by the change of the tumor volume, edema, metabolic indices, etc. through a radiological examination preoperatively [8–16]. There are several prediction models developed to distinguish good responders from others for patients with HOS. ^18^F-FDG PET/CT has a good performance in predicting the pathologic response, whereas its cost is high [12–16]. MRI has a certain predictive effect, but the accuracy of the judgment is not high enough [8–11]. According to Holscher et al., increase of tumor volume indicates poor histopathologic response (sensitivity 89%, specificity 73%) [17]. Decreased or unchanged tumor volume and a decrease in edema were poor predictors of good histopathologic response (predictive values, 56–62%) [8]. While, an increase in the size of areas of low signal intensity, and a decrease in joint effusion occurred independently of histopathologic response in almost half of the patients [8]. Most previous studies have focused on qualitative description of medical images, which may have limitations in predicting chemotherapeutic responses. Moreover, most of them used a mean value to depict whole tumors, potentially overlooking tumor heterogeneity.

Radiomics, which involves extracting quantitative features from medical images, is capable of generating imaging biomarkers as decision support tools for clinical practice [18–26]. The traditional radiomics method utilizes single-phase medical images for evaluation or prediction, which neglects the tumor change during treatment or following up. The delta-radiomics concept [18], which employs the change in radiomic features during or after treatment to instruct clinical decisions, may be more suitable for evaluation of tumor response of treatment. The delta-radiomics method has been shown to be predictive in prognoses and metastases in previous studies. Carvalho et al. found the delta-radiomic features of PET images predictive of the overall survival in non-small cell lung cancer patients [27]. Fave et al. suggested the delta-radiomic features from CT images after radiation therapy may be indicators of tumor response in non-small cell lung cancer patients [28]. As pretreatment CT is associated with responses to NCT while posttreatment CT directly reflects the posttreatment status, a radiomics model combining pre- and posttreatment CT data may potentially predict pathologic response with accuracy. To the best of our knowledge, no previous studies have explored the capability of delta-radiomic features of CT in tumor response evaluation for HOS patients. Delta-radiomics may offer better clinical decision support and have enormous potential for precision medicine.

Thus, in our retrospective study, we aim to develop and validate a delta-radiomics nomogram in evaluating pathologic responses after NCT in patients with HOS. Consistent with clinical practice, our work combined pre- and posttreatment CT data to noninvasively evaluate the outcomes of patients and identify the non-good response HOS patients.

## Methods

### Patients

This retrospective study reviewed the medical images and clinical records of all patients with osteosarcoma registered at our hospital between November 2013 and November 2017. This study was approved by the Institutional Research Ethics Board and the informed consent requirement was waived. This study was conducted according to the Declaration of Helsinki. All patients included in the study met the following criteria: they had undergone NCT and subsequent surgical resections; they had diagnostic CTs before and after chemotherapy, and we had access to their complete histologic information. All patients were diagnosed with HOS according to World Health Organization (WHO) Classification of Tumors of Soft Tissue and Bone, they have many subtypes such as osteoblastic, chondroblastic, fibroblastic, telangiectatic, small cell and high-grade surface (juxtacortical high grade) [29]. All patients had diagnostic CTs of tumor site before and after chemotherapy, with an interval of 9 to 11 weeks. Lung CT was performed before, during, and after chemotherapy to determine the presence of pulmonary metastasis, with intervals ranging from 4 to 11 weeks. Each patient received emission computed tomography (ECT) pre-chemotherapy to evaluate the primary lesion and potential metastatic foci. Of the 261 patients diagnosed with HOS at our institution, 191 fulfilled these criteria. Additional file 1: Figure S1 shows the patient recruitment pathway. The clinical factors of age, gender, tumor location, tumor stage, pathologic subtype, type of surgery, new pulmonary metastasis and chemotherapy regimens were acquired for the study by reviewing patients’ medical records. The patients’ data were divided into training (*n* = 137) and validation (*n* = 54) datasets according to patients’ admission times. The data of patients admitted after November 2016 were used for validating the developed model.

### Chemotherapy and histologic analysis

All patients received neoadjuvant chemotherapy followed by surgical resection. The treatment protocol and schedule followed the National Comprehensive Cancer Network guidelines. The conventional three-drug regimen, (Regimen-1) consisting of methotrexate, cisplatin and doxorubicin, was followed by a subsequent surgical resection. The patients who suffered severe liver dysfunction or other adverse reactions after the administration of methotrexate during the first cycle of NCT received Regimen-2 treatment consisting of methotrexate, ifosfamide, cisplatin and doxorubicin preoperatively. Regimen-3, consisting of methotrexate, ifosfamide, cisplatin and doxorubicin, was used in cases of tumor progression or new lung metastasis during the first chemotherapy cycle. The total duration of NCT was at least 8–10 weeks. The complete schedules for these regimens are shown in Additional file 1: Figure S2.

We analyzed the histologic response to preoperative chemotherapy using the method of Bacci et al. by two experienced pathologists [7]. Tumor necrosis percentages graded as III and IV (tumor necrosis≥90%) indicated a pathological good response (pGR), while those graded as I and II (necrosis < 90%) indicated a non-pGR [6].

### Technical parameters for CT image acquisition

**Fig. 1:**
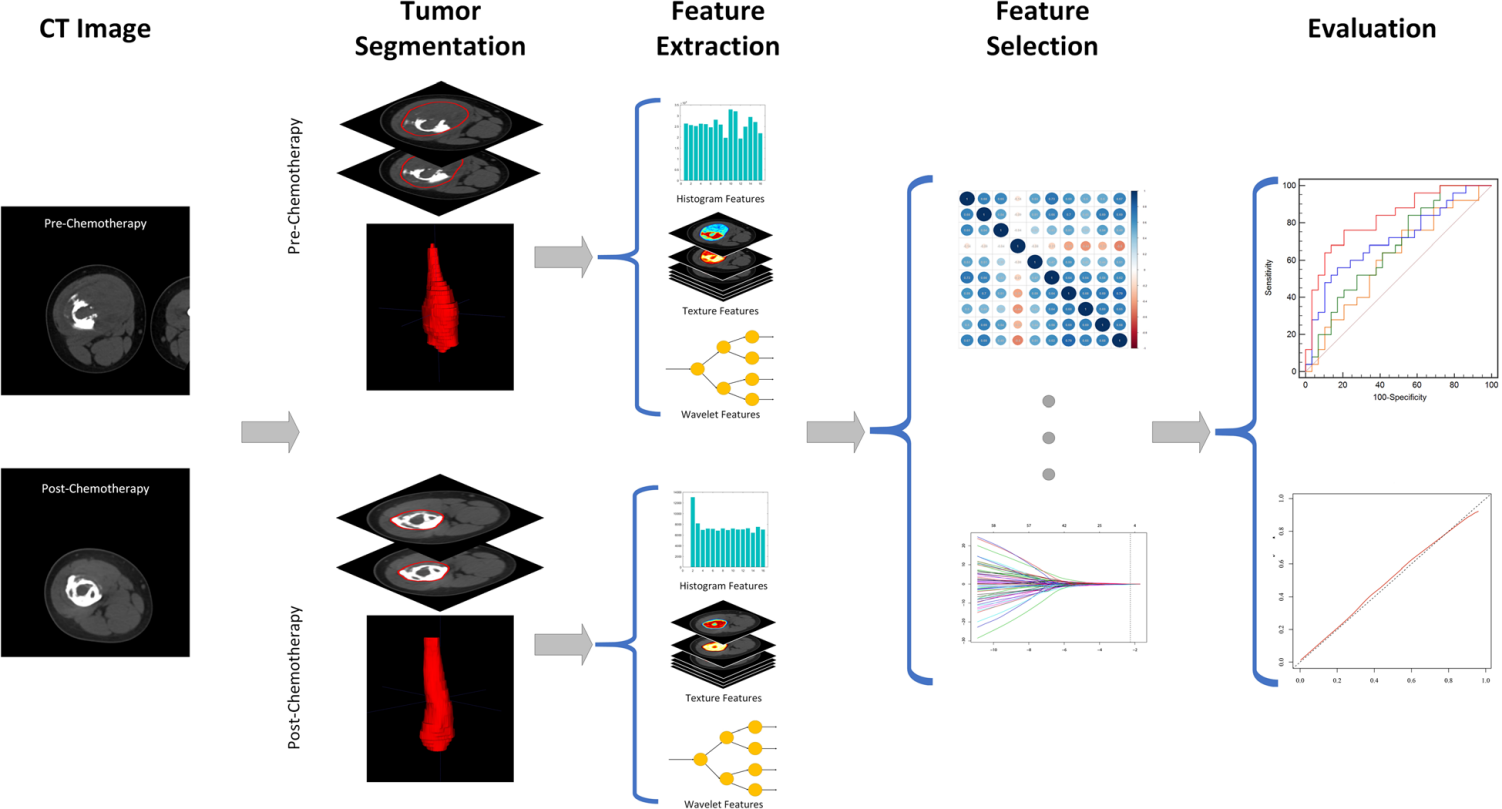
The radiomics schematic depiction of this study

Fig. 1 depicts the schematic of our study. The pretreatment and posttreatment CT scans were acquired on one of the 40-slice, 64-slice and 128-slice spiral CT scanners (Siemens Medical Systems, Philips Medical Systems, Toshiba Medical Systems) in our institution. The CT scans were with one of the four tube voltages (80kVp, 100kVp, 120kVp, 140kVp) and tube current of 200–500 effective mAs, for different patients. The CT images were reconstructed into a matrix of 512 × 512. The reconstruction FOV varied from 132.5 to 475 mm, corresponding to pixel sizes ranging from 0.2588 to 0.9277 mm and slice thickness of 4 or 5 mm, according to the tumor volume circumstances (pelvis, femur, tibia, humerus and extremity).

### Tumor segmentation

We used the pretreatment and posttreatment CT scans to quantify tumor heterogeneity in this study. The detailed imaging parameters are listed above. The 3-dimensional tumor regions were contoured from both the pretreatment and posttreatment CT scans as the region of interest (ROI) for this study. Two experienced orthopedists performed the tumor segmentation using the open-source software ITK-SNAP as reported [22]. The contours were then checked by a radiologist to ensure their accuracy and were modified if necessary. Both orthopedists and radiologists agreed upon all the ROIs for this study. The tumors in the training cohort were segmented by Orthopedist-1 twice and Orthopedist-2 once, separately. The two sets of radiomic features based on the segmentation of Orthopedist-1 were used for intra-observer reproducibility test and model training. The radiomic features based on the segmentations of Orthopedist-1 and Orthopedist-2 were used for inter-observer reproducibility test. Tumors in the validation cohort were segmented by Orthopedist-1 to test the prediction power of the trained model. For cases where the boundary of soft tissue mass is unclear on the CT, the patient’s MRI image was referenced during the segmentation.

### Feature extraction

Feature extraction was performed using open-source Radiomics packages by Vallières M. et al., [30, 31] which were implanted onto Matlab software (Matlab 2016, MathWorks). All CT scan images were resampled to 1 mm resolution on all three directions to standardize the voxel size across the patients [32]. The radiomic features that characterize the intensity and texture of the tumors were extracted for each region. The wavelet transformation was performed on the tumor region at eight directions to fully quantify the tumor in multiple dimensions.

The intensity features measured the gray level distribution in the tumor region and were quantified as mean, energy, entropy, variance, skewness, kurtosis and uniformity. The texture features characterized the tumor’s texture properties based on the gray-level co-occurrence matrix (GLCM, *n* = 22), the gray-level size-zone matrix (GLSZM, *n* = 13), the gray-level run-length matrix (GLRLM, n = 13) and the neighborhood gray-tone-difference matrix (NGTDM, *n* = 5). In summary, 7 intensity features and 53 texture features were extracted from each ROI.

The wavelet-based features were derived by performing texture analysis on the wavelet transformed tumor region on the x, y and z-axes, similar to Fourier analysis. The wavelet transformation decomposed the tumor region images into high-frequency components (H) or low-frequency components (L) at the three directions. Eight categories of wavelet features were acquired and labeled as HHH, HHL, HLH, LHH, LLL, LLH, LHL, HLL based on their different decomposition order. For example, the HLH category features are the texture features derived from the tumor region after a high pass filter on the x-direction, a low pass filter decomposition on the y-direction and a high-frequency wavelet decomposition on the z-direction. For each category, the intensity and texture features were calculated, resulting in 480 wavelet-based radiomic features for each ROI.

The radiomic features were extracted from the tumor regions on pre-chemotherapy CTs (pre-chemotherapy radiomic features, PRE-RFs) and post-chemotherapy CTs (post-chemotherapy radiomic features, PST-RFs), respectively. The delta-CT features (Delta-RFs) were defined as the change of radiomic feature after chemotherapy and calculated by subtracting PRE_RFs from PST_RFs, as shown in Eq. 1.

1 $$ \mathrm{Delta}-\mathrm{RF}=\mathrm{PST}-\mathrm{RF}-\mathrm{PRE}-\mathrm{RF} $$

### Feature selection and Radiomics signature building

The training datasets were used for feature selection and radiomics signature building. The radiomic features which were robust in both the inter-observer and intra-observer reproducibility tests were used for further analysis. The interclass correlation coefficient (ICC) was used to evaluate the reproducibility of radiomic features across different segmentations and robust radiomic features were defined as those with ICCs of more than 0.75 [33]. To exclude highly redundant radiomic features, a correlation matrix was constructed using pair-wise Pearson correlation analysis [34]. The features that showed high correlation (correlation coefficient > 0.95) with other features were then excluded from the analysis.

We used the Mann-Whitney U test to assess the ability of the delta-radiomic features in differentiating pGR patients from non-pGR patients. The radiomic features with statistical significance between the pGR group and the non-pGR group were left for further analysis.

The least absolute shrinkage and selection operator (LASSO) regression was used to perform the radiomic features selection in the training dataset. The LASSO method was usually implanted in the feature selection of high-dimensional data by minimizing classification errors, tuning the sum of absolute values of the feature coefficients to be no more than a parameter λ [35]. The coefficients of some features are reduced to zero by tuning the λ. Only features with non-zero coefficients were selected in the final model. A radiomics signature was then built by summing the features multiplied by their coefficient. Ten-fold cross-validation was used in determining the tuning parameter λ. The λ value that resulted in the least binomial deviance in the ten-fold cross validation was selected in this study. The receiver operating characteristic (ROC) curve and the area under the ROC curve (AUC) were used to assess the predictive accuracy of the developed delta-radiomics signature (**Radiomics Signature I**).

To show the unique predictive value of Delta-RFs, we also compare the prediction performance of delta-radiomics signature with the radiomics signatures constructed using only PRE-RFs (**Radiomics Signature II**), PST-RFs (**Radiomics Signature III**) respectively and combining PRE-RFs and PST-RFs (**Radiomics Signature IV**). The radiomics signature II, III, IV were constructed using the same analysis workflow with Delta-RFs.

### Delta Radiomics Nomogram construction

The multivariable logistic regression method was used for examining the prediction value of combining radiomics and clinical features. The backward elimination method was used in selecting the optimum feature subset [36]. The delta-radiomics nomogram was constructed based on the final model. The developed delta-radiomics signature and nomogram were then validated on the validation dataset.

### Statistical analysis

Chi-square and Mann-Whitney U tests were used for categorical and continuous clinical factors between the two groups, respectively. The *p* values of multiple comparison Mann-Whitney U test were corrected using the false discovery rate method. The optimal cutoff was calculated by Youden index in the ROC curve analysis. The calibration curve was used to assess the predictive accuracy of the developed nomogram. Decision curve analysis (DCA) was conducted to evaluate whether the nomogram was sufficiently robust for clinical practice [37]. A value of *p* < 0.05 was considered statistically significant. All *p* values were two-sided in this study. All statistical analysis was performed with R software (version 3.4.1; http://www.Rproject.org). The LASSO logistic regression analysis was performed using the “glmnet” package. The nomogram was plotted based on the “rms” package. The ROC curve was plotted using MedCalc 15.2.2 (MedCalc Inc., Mariakerke, Belgium).

## Results

### Patient characteristics

Patient characteristics in the training and validation sets are detailed in Table 1 and Additional file 1: Table S1. There were no significant differences between the two sets in chemotherapeutic response (pGR and non-pGR), age, gender, tumor volume, tumor location, tumor stage, pathologic subtype, type of surgery, new pulmonary metastasis and chemotherapy regimens. The Non-pGR rates were 58.4 and 53.7% in the training and validation cohorts, respectively, and there were no significant differences between them (*p* = 0.6691).

**Table 1:** Characteristics at time of diagnosis in patients with high-grade osteosarcoma

Characteristic	Training cohort (*n* = 137)		*P*	Independent validation cohort (*n* = 54)		*P*
	pGR (*n* = 57)	Non-pGR (*n* = 80)		pGR (*n* = 25)	Non-pGR (*n* = 29)	
Age, years						
Median (range)	16 (4.6–43)	14 (4–46)	0.3939	15 (8–39)	18 (7–44)	0.6123
≤ 15 y	27	45		13	12	
> 15 y	30	35		12	17	
Gender			1			0.5852
Male	34	47		14	13	
Female	23	33		11	16	
Location of primary tumor			0.3447			0.8041
Humerus	11	8		3	3	
Femur	27	45		14	17	
Tibia and fibula	17	20		8	8	
Radius and ulna	1	2		0	0	
Others	1	5		0	1	
Stage at diagnosis			1			0.3062
Localized	47	66		20	27	
Metastatic	10	14		5	2	
Pathologic subtype			0.3055			0.332
Osteoblastic	46	55		20	19	
Chondroblastic	3	13		1	5	
Fibroblastic	4	4		4	4	
Telangiectatic	3	5		0	1	
Others	1	3		0	0	
Type of surgery			0.02487*			1
Limb salvage	55	66		24	27	
Amputation	2	14		1	2	
New pulmonary metastasis			1			0.9402
Yes	2	4		1	0	
No	55	76		24	29	
Chemotherapy regimens			0.7224			0.4406
1MTX, DDP and ADM	42	58		17	22	
2MTX, IFO,DDP and ADM	12	15		8	6	
3MTX,IFO, DDP and ADM	3	7		0	1	
Radiomics score	4.4E-4(−1.1–0.72)	−0.55(−2.9–0.32)	2.1E-14	0.030(−0.58–0.71)	−0.31(−2.1–0.34)	2.4E-5

Note: Individual clinical factors were analyzed for significant differences using a nonparametric test. *P < 0.05 indicates a significant difference. Ages and radiomics scores are represented as [Median (range)]. Methotrexate (*MTX*), Ifosfamide (*IFO*), Cisplatin (*DDP*) and Doxorubicin (*ADM*)

### Features selection and Radiomics signature building

**Fig. 2:**
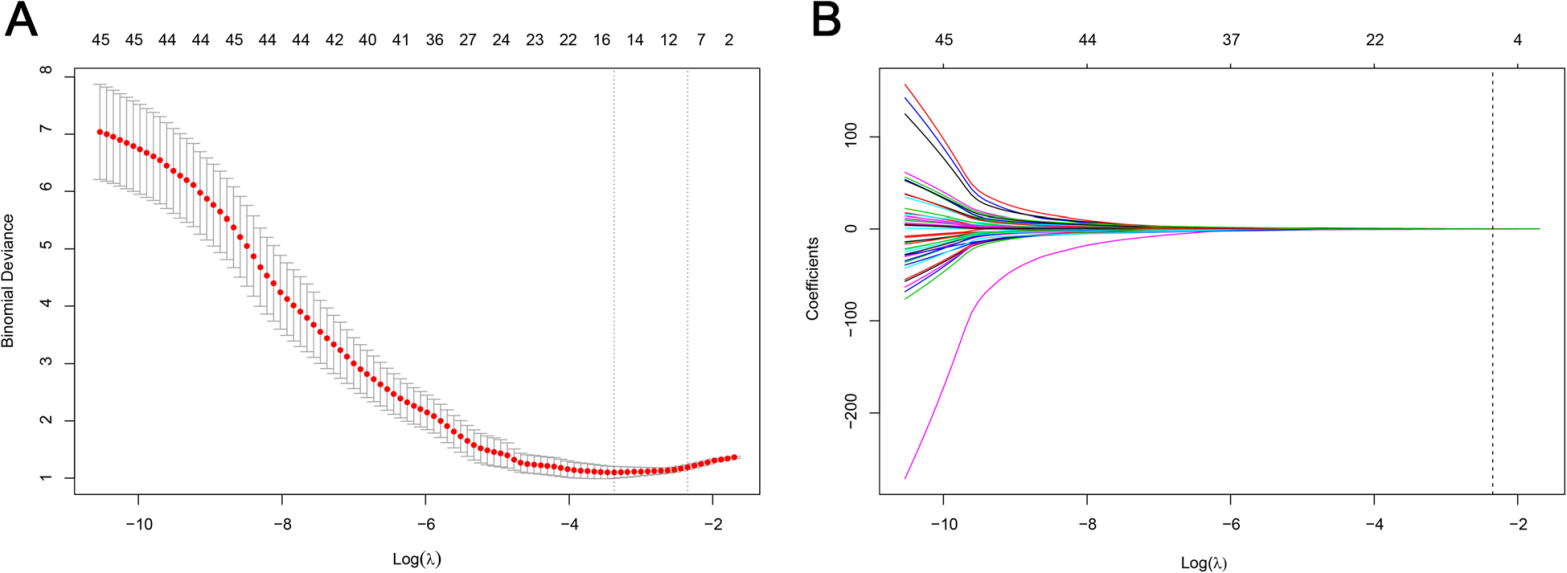
Ten-fold cross-validation results using the LASSO method. (**a**) The binomial deviance metrics (the y-axis) were plotted against log(λ) (the bottom x-axis). The top x-axis indicates the number of predictors with the given log(λ). Red dots indicate the average AUC for each model at the given λ, and vertical bars through the red dots show the upper and lower values of the binomial deviance in the cross-validation process. The vertical black lines define the optimal λ, where the model provides its best fit to the data. As a result, the optimal λ of 0.1047237, with log(λ) = − 2.256430, was selected. (**b**) The LASSO coefficient profiles of the 45 radiomic features are depicted. The vertical line was plotted at the given λ. For the optimal λ, eight features with non-zero coefficients were selected

In total, 540 radiomic features were extracted from tumor lesions on the pre-treatment and post-treatment CT scans, respectively, resulting in 540 Delta-RFs. A total of 382 Delta-RFs were robust in both the intra-observer analysis and inter-observer analysis. Then, 198 Delta-RFs with a correlation coefficient < 0.95 were selected for further analysis. By applying the Mann-Whitney test on the pre-selected features, 45 instructive Delta-RFs showed significant differences between the pGR group and the non-pGR group with a *p*-value < 0.05 and are shown in Additional file 1: Figure S3. Through the LASSO logistic regression analysis, eight Delta-RFs were selected (shown in Fig. 2). All the selected Delta-RFs were reproducible in the intra−/inter-observer test with ICC of more than 0.8. The detailed ICC values of selected Delta-RFs were shown in Additional file 1: Table S2. Based on the eight Delta-RFs and their coefficients, a delta-radiomics signature was calculated for each patient. The delta-radiomics signature formula is given below.

2 $$ \mathrm{Delta}\ \mathrm{Radiomics}\ \mathrm{Signature}=0.040868419\times \Delta  \mathrm{variance}-0.112921064\times \Delta  \mathrm{LLL}\_\mathrm{GLCM}\_\mathrm{corrp}-0.131641870\times \Delta  \mathrm{LLH}\_\mathrm{Entropy}-0.215106590\times \Delta  \mathrm{LLH}\_\mathrm{GLSZM}\_\mathrm{GLN}-0.162624738\times \Delta  \mathrm{LHH}\_\mathrm{GLSZM}\_\mathrm{ZSN}-0.049041868\times \Delta  \mathrm{HHL}\_\mathrm{GLCM}\_\mathrm{corrm}+0.042748856\times \Delta  \mathrm{HHH}\_\mathrm{GLSZM}\_\mathrm{SZE}+0.001226972\times \Delta  \mathrm{HHH}\_\mathrm{GLSZM}\_\mathrm{SZHGE} $$

### Performance of the Radiomics signature

**Fig. 3:**
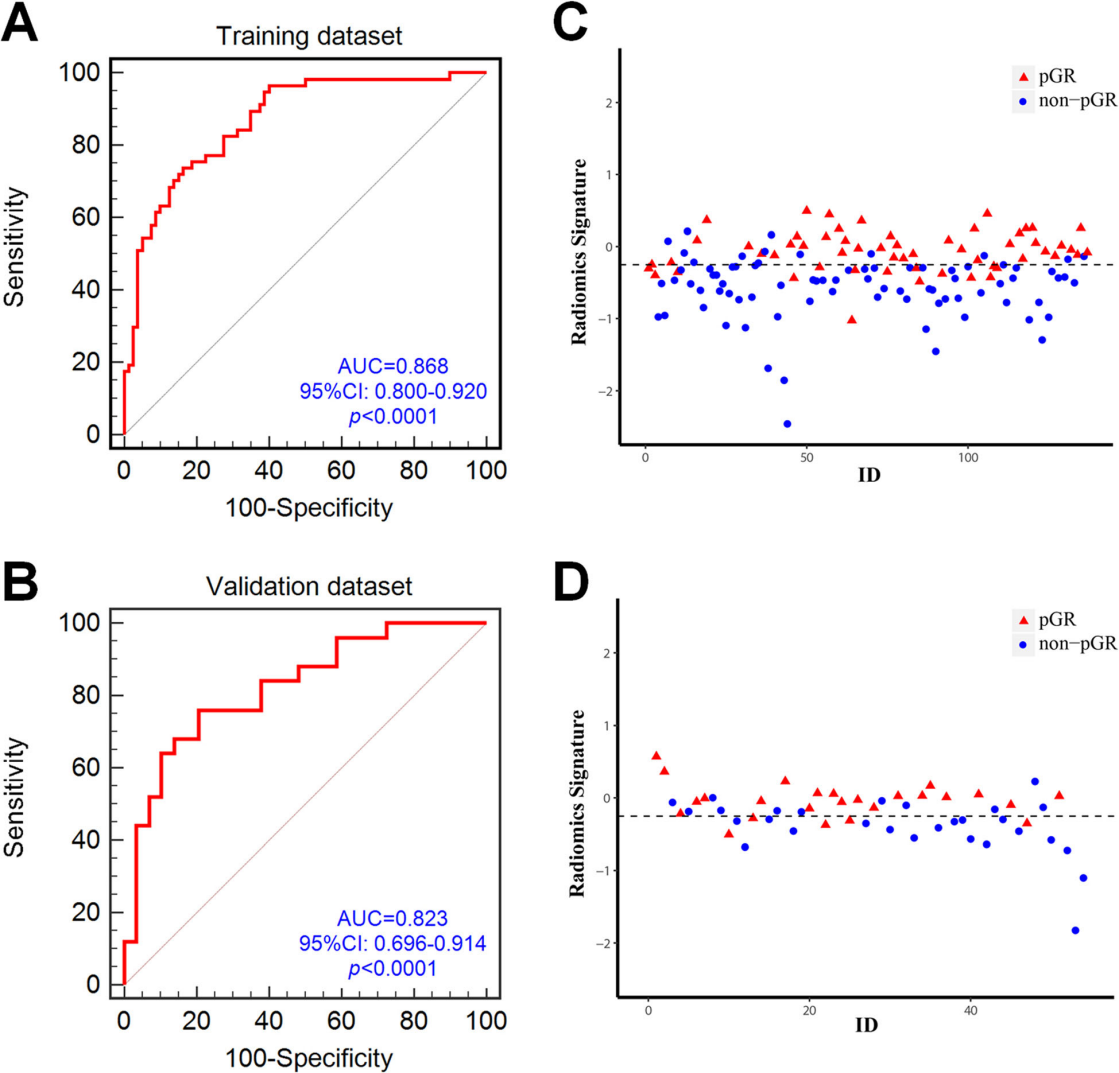
The predictive performance of the radiomics signature for each patient in training (**a**) and validation (**b**) sets (95% CI, 95% confidence interval; AUC, area under curve). The radiomics signature for each patient in training (**c**) and validation (**d**) sets. Blue dots show signature values for non-pGR patients, while red triangles indicate values for pGR patients. The dotted line shows the best cutoff values calculated by Youden test, which is − 0.251 for the training dataset

The delta-radiomics signature was significantly different between pGR and non-pGR patients in both the training and the validation datasets (both *p* < 0.0001). The ROC analysis exhibited good prediction value of the developed delta-radiomics signature in this study with an AUC of 0.868 in the training dataset and AUC of 0.823 in the validation dataset **(**Fig. 3 a, b**)**. The delta-radiomics signature values of patients are shown in Fig. 3 c, d. Compared with radiomics signature II, III, IV, the delta-radiomics signature shows the highest AUC in both the training and validation datasets, which is illustrated in Additional file 1: Figure S4.

### Radiomics Nomogram building and evaluation

**Fig. 4:**
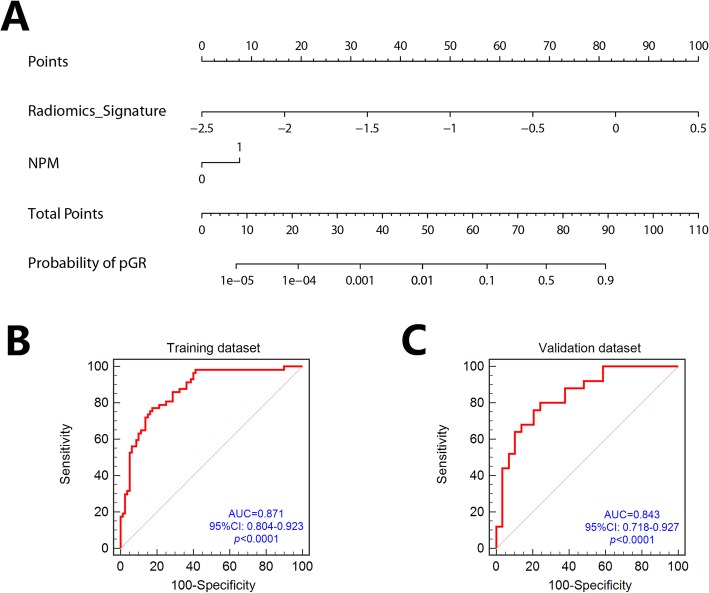
(**a**) The radiomics nomogram incorporating the radiomics signature and NPM. The ROC curves for the radiomics nomogram in training (**b**) and validation (**c**) sets

**Fig. 5:**
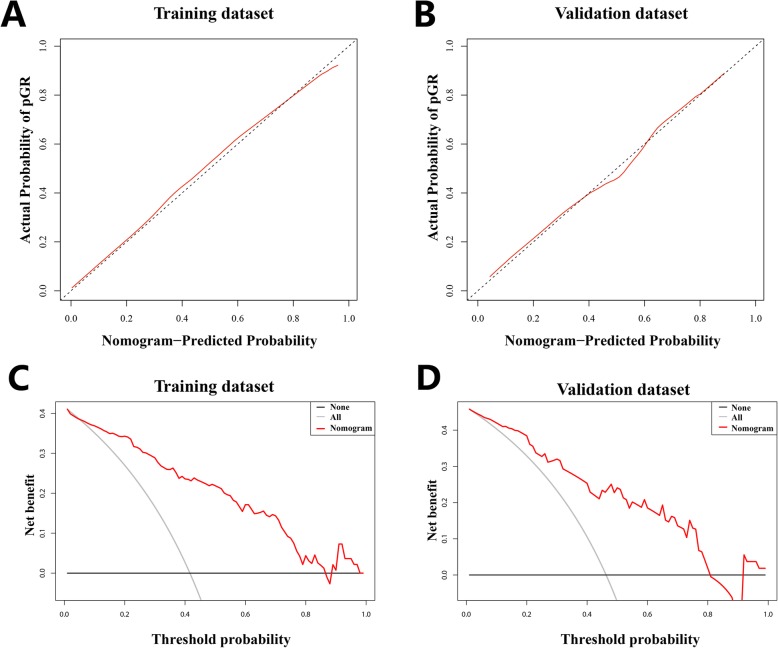
The calibration curve of the developed radiomics nomogram in the training dataset (**a**) and validation dataset (**b**). Calibration curves depict the calibration of each model according to the agreement between the predicted probability of pathologic good response (pGR) and actual outcomes of the pGR rate. The y-axis represents the actual rate of pGR. The x-axis represents the predicted probability of pGR. The diagonal black line represents an ideal prediction. The red line represents the performance of the radiomics nomogram, of which a closer fit to the diagonal black line represents a better prediction. Decision curve analysis (DCA) for the radiomics nomogram in both training (**c**) and validation cohorts (**d**). The y-axis indicates the net benefit; x-axis indicates threshold probability. The red line represents the radiomics nomogram. The gray line represents the hypothesis that all patients showed pGR. The black line represents the hypothesis that no patients showed pGR

To build the final model in the backward search process, we combined the delta-radiomics signature and new pulmonary metastases (NPM) during chemotherapy. We built a radiomics nomogram which was based on the multivariable logistical regression model using the delta-radiomics signature and NPM as shown in Fig. 4 a. The ROC analysis result demonstrated the improved prediction value of the developed radiomics nomogram. After incorporating NPM in the prediction model, the AUC in the training and validation datasets increased to 0.871 and 0.843, respectively (Fig. 4 b, c). The calibration curve analysis also indicated the high predictive accuracy of the developed radiomics nomogram with a mean absolute error of 0.015 and 0.017 in the training and validation datasets, respectively (Fig. 5 a, b). DCAs for the radiomics nomogram in the training and validation datasets are shown in Fig. 5 c and d. The decision curve showed relatively good performance for the model according to clinical application. When the threshold probability of pGR is between 0 and 0.84 in the training set or between 0 and 0.81 in the validation set, using the radiomics nomogram to predict pGR adds more benefit than treating either all or no patients.

## Discussion

In this present study, we developed and validated a diagnostic, delta-radiomics signature-based nomogram for the noninvasive, preoperative individualized evaluation of chemotherapeutic response in patients with HOS. The radiomics signature successfully differentiated patients according to their chemotherapeutic response. The easy-to-use nomogram facilitates the noninvasive individualized evaluation of a patient’s chemotherapeutic response and therefore provides an effective tool for clinical decision-making.

The accurate identification of non-pGR patients using visual judgment (conventional CT, MRI) remains challenging in clinical practice. Methods using ^18^F-FDG PET/CT or ^18^F-FDG PET/CT combining MRI may have a good performance. Maximum standardized uptake value (SUVmax), metabolic tumor volume (MTV) and total lesion glycolysis (TLG) that derived from 18F-FDG PET/CT or 18F-FDG PET/CT combining MRI were associated with histologic response and may have a good performance in differentiating histologic response [13, 14, 16]. However, they are relatively expensive and not easy to popularize. Radiomics analysis integrates high-dimensional imaging features, which are difficult to detect visually when evaluating the non-pGR. Our proposed delta-radiomics nomogram based on these imaging features showed a better performance than previously reported methods. It can therefore be helpful in clinical decision-making as it provides oncologists with a potential quantitative tool for individualized non-pGR prediction.

To use our proposed radiomics model, radiologists must first delineate the regions of interest (ROI) on pre- and post-chemotherapeutic CT scans, after which the model allows for the calculation of the probability of non-pGR for each individual patient. Oncologists can then consider various factors, including the calculated probability of non-pGR and other retrievable clinical information, as well as their own clinical experience, to make a comprehensive judgment on whether to modify the treatment strategy.

Previously, there have been a few studies evaluating the prognostic value of ^18^F-FDG PET/CT and MRI in assessing the chemotherapy outcome for HOS [8–13, 15, 16]. Imaging radiomics has been studied in predicting the pathologic response after preoperative chemoradiotherapy for locally advanced rectal cancer [38]. Radiomics signature-based nomograms are currently being used in the prediction of pathological responses to chemoradiotherapy or chemotherapy in certain cancers [39, 40]. Although radiomics signature-based nomograms or imaging radiomics has formerly been used in survival prediction and the differentiation of pulmonary metastases from non-metastatic nodules in osteosarcoma [22, 41]. To the best of our knowledge, this is the first study evaluating the pathological response after chemotherapy for HOS using a radiomics nomogram.

We evaluated the ability of texture features in differentiating non-pGR patients with HOS. The texture analysis was previously used for tissue classification in medical images [42], showing the capability of texture analysis in quantifying tumor heterogeneity. For the construction of the delta-radiomics signature, 540 candidate delta-radiomic features were reduced to an 8-feature combined signature by the LASSO method. The feature selection process reduced the over-fitting error and the impact of the noise and random error [42], making the developed radiomics model more robust and stable.

The radiomics model we proposed achieved a relatively high negative predictive value and positive predictive value in both the training and validation cohorts. The high negative predictive value in this study indicated that the non-pGR evaluation of the proposed model was reliable. Thus, oncologists may potentially adjust the chemotherapy regimen or intensify the chemotherapy. In some cases, surgeons may even choose aggressive surgery. Conversely, the high positive predictive value suggests that our model can accurately enable oncologists to screen out pGR patients.

Recently, many studies have used MRI to predict a pathological response, and the tumors they evaluated were mainly soft tissues. Diffusion-weighted imaging is considered to have strong potential in predicting the responses to chemoradiotherapy in patients with locally advanced rectal cancer [37, 43]. To be different, as HOS, evaluated in this study, mainly occurs in the skeleton, CT scans have greater advantages in evaluating bone destruction and osteoid production comparing to MRI. In addition, CT is a conventional, highly popular examination at low cost. However, it is insufficient to evaluate edema and metabolic levels when compared with MRI and PET. Therefore, if CT scanning were combined with MRI and PET, the prediction accuracy would likely be higher. A further study combining CT, MRI and PET images together would most probably achieve better prediction accuracy.

Changes in tumor volume have previously been suggested as a prediction factor to the pathologic response by several authors, who reported that the sequestration and disappearance of a tumor may be correlated with a good pathologic response. Conversely, the increase or no change in tumor volume suggests a poor response to chemotherapy. However, the situation might be quite different in osteosarcoma, a tumor that does not shrink to a great extent after neoadjuvant chemotherapy [12]. Nevertheless, in some cases, the tumor may undergo necrosis or liquefaction and become avascular or cystic, without a significant change in tumor size. Some may even have increased in size. The accuracy of the judgment based on changes in tumor volume in these cases is not high enough. The voxel-wise analysis could provide additional information, comparing conventional volume-averaged analysis in assessing the therapeutic response. Therefore, it is an important tool to interrogate tumor pathological response.

In the present study, we use the delta-radiomics method. A clinician could request the radiomic analysis of a patient based on their diagnostic CT images, potentially enabling an improved early chemotherapeutic response evaluation, improved clinical decision-making and, consequently, a better prognosis [18].

The present study has some **limitations.** First, we retrospectively analyzed only the patients who met the inclusion criteria, which may have been prone to selection bias. Second, the sample size of the cohort was relatively small. Third, all the patients were from a single institution. The performance of the model may differ when used with multi-centric datasets with different parameters. Further, better-controlled prospective studies in multi-centric settings with a larger sample of patients would be required to validate the reliability and reproducibility of our proposed radiomics model.

## Conclusions

In conclusion, using pre- and posttreatment CT data, we developed a delta-radiomics nomogram with excellent performance for an individualized, noninvasive pathologic response evaluation after NCT. This model may help tailor appropriate treatment decisions for HOS patients.

## Supplementary information

**Additional file 1: Table S1** Patient characteristics’ distribution in the training and validation datasets. **Table S2:** Interclass correlation coefficient (ICC) values of selected delta-radiomic features in the intra-observer and inter-observer reproducibility test. **Fig. S1.** Recruitment pathway for patients. **Fig. S2.** Regimens of preoperative treatment protocols of neoadjuvant chemotherapy. MTX: methotrexate; DDP: cisplatin; ADM: doxorubicin; IFO: ifosfamide. **Fig. S3.** Heatmap for instructive radiomic features in the training set. The x-axis indicates different patients. The y-axis indicates different radiomics features. The color in the box shows the expression level of radiomic features. **Fig. S4.** The predictive performance of the radiomic signature from four kinds of data for each patient in training (A) and validation (B) sets (AUC, area under curve)

## Data Availability

The datasets used and analysed during the current study are available from the corresponding author on reasonable request.

## Abbreviations

AUC: Area under curve
CI: Confidence interval
DCA: Decision curve analysis
HOS: High-grade osteosarcoma
LASSO: Least absolute shrinkage and selection operator
NCT: Neoadjuvant chemotherapy
NPM: New pulmonary metastases
pGR: Pathologic good response
ROC: Receiver operating characteristic
ROI: Region of interest
WHO: World Health Organization
