# Supplementary material for: A Delta-radiomics model for preoperative evaluation of Neoadjuvant chemotherapy response in high-grade osteosarcoma
Source: Cancer Imaging. 2020 Jan 14;20:7. doi: 10.1186/s40644-019-0283-8 (PMC6958668; doi:10.1186/s40644-019-0283-8)
Supplement: Supplementary file 1 — Additional file 1: Table S1 Patient characteristics’ distribution in the training and validation datasets. Table S2: Interclass correlation coefficient (ICC) values of selected delta-radiomic features in the intra-observer and inter-observer reproducibility test. Fig. S1. Recruitment pathway for patients. Fig. S2. Regimens of preoperative treatment protocols of neoadjuvant chemotherapy. MTX: methotrexate; DDP: cisplatin; ADM: doxorubicin; IFO: ifosfamide. Fig. S3. Heatmap for instructive radiomic features in the training set. The x-axis indicates different patients. The y-axis indicates different radiomics features. The color in the box shows the expression level of radiomic features. Fig. S4. The predictive performance of the radiomic signature from four kinds of data for each patient in training (A) and validation (B) sets (AUC, area under curve) [file 40644_2019_283_MOESM1_ESM.docx]

Supplementary Material

# Supplementary Data

**I. Supplementary Figure S1** Recruitment pathway for patients.


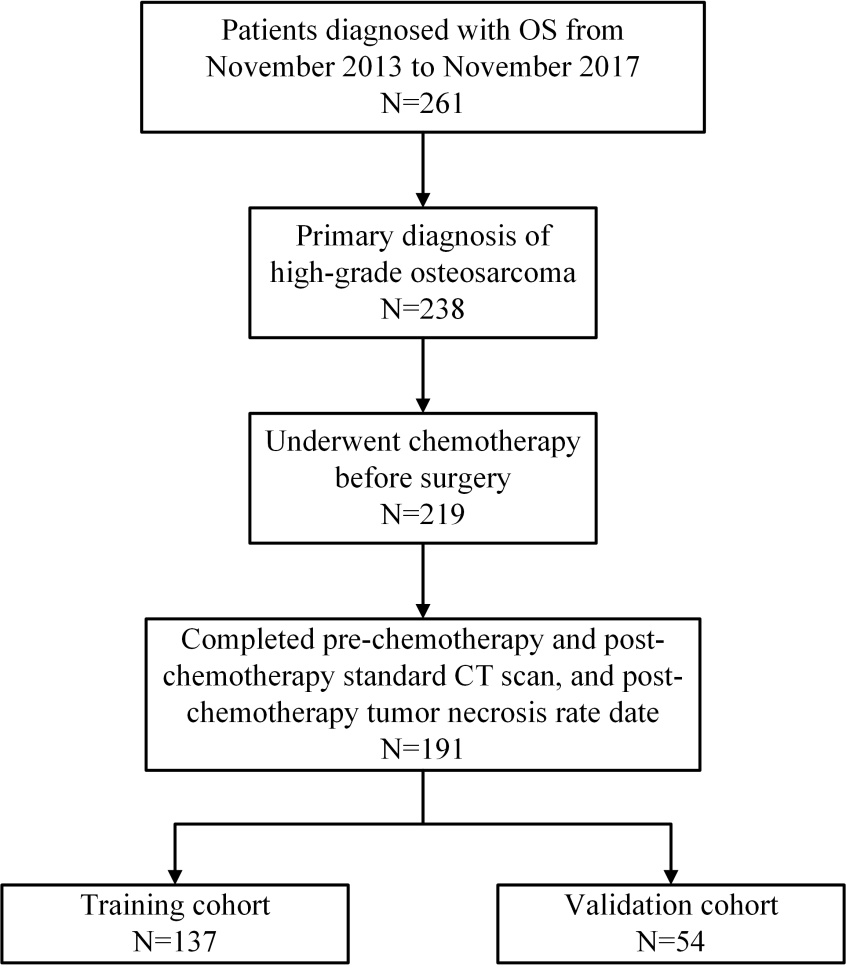


**Figure S1.** Recruitment pathway for patients.

**II. Supplementary Figure S2** Neoadjuvant chemotherapy regimens.

**
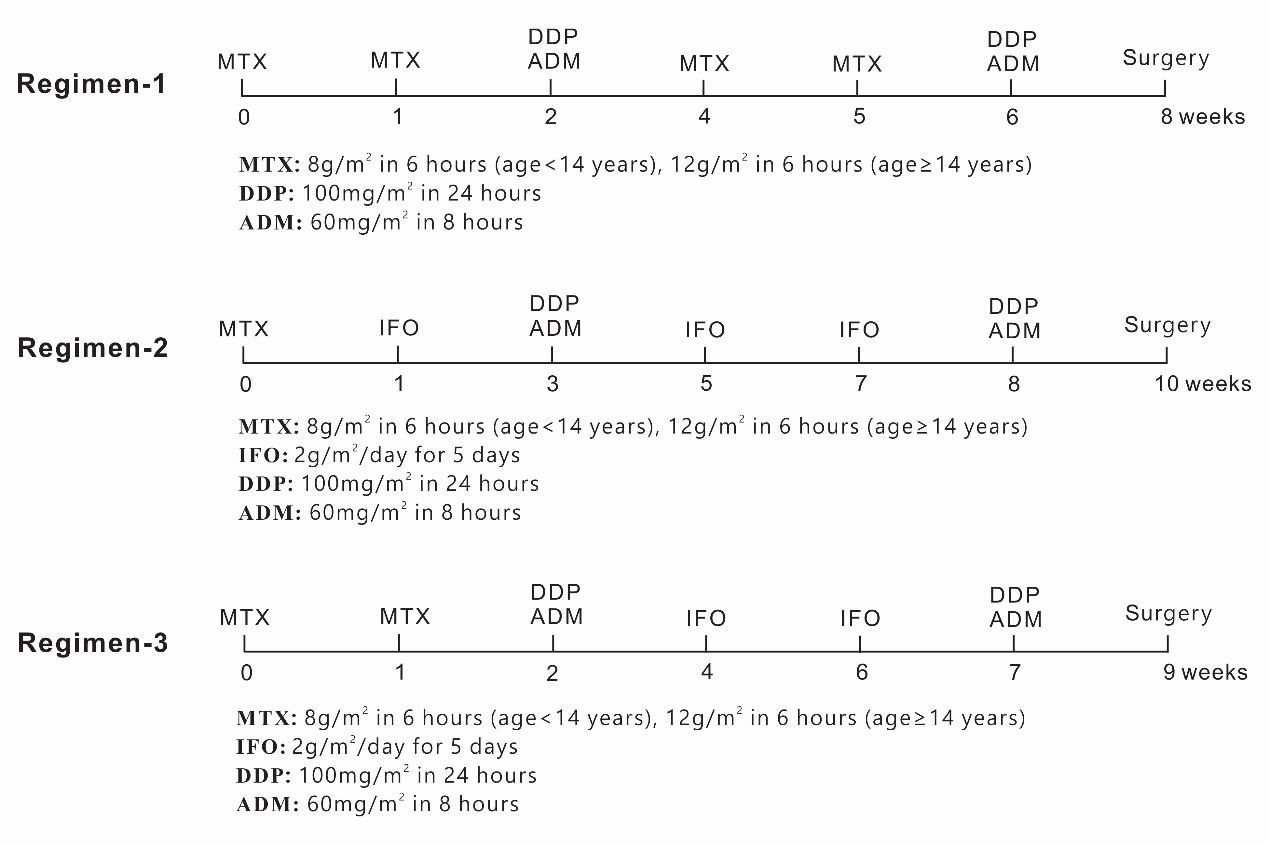
**

**Figure S2.** Regimens of preoperative treatment protocols of neoadjuvant chemotherapy. MTX: methotrexate; DDP: cisplatin; ADM: doxorubicin; IFO: ifosfamide.

**III. Table S1** Patient characteristics’ distribution in the training and validation datasets

| Characteristics | Training dataset | Validation dataset | p |
| --- | --- | --- | --- |
| Pathological Response |  |  | 0.6691 |
| pGR | 57 | 25 |  |
| non-pGR | 80 | 29 |  |
| Age |  |  | 0.5363 |
| ≤15 y | 72 | 25 |  |
| >15 y | 65 | 29 |  |
| Gender |  |  | 0.3254 |
| Male | 81 | 27 |  |
| Female | 56 | 27 |  |
| Location of primary tumor |  |  | 0.6721 |
| Humerus | 19 | 6 |  |
| Femur | 72 | 31 |  |
| Tibia and fibula | 37 | 16 |  |
| Radius and ulna | 3 | 0 |  |
| Others | 6 | 1 |  |
| Stage at diagnosis |  |  | 0.5816 |
| Localized | 113 | 47 |  |
| Metastatic | 24 | 7 |  |
| Pathologic subtype |  |  | 0.1567 |
| Osteoblastic | 101 | 39 |  |
| Chondroblastic | 16 | 6 |  |
| Fibroblastic | 8 | 8 |  |
| Telangiectatic | 8 | 1 |  |
| Others | 4 | 0 |  |
| Type of surgery |  |  | 0.315 |
| Limb salvage | 121 | 51 |  |
| Amputation | 16 | 3 |  |
| New pulmonary metastasis |  |  | 0.6821 |
| Yes | 6 | 1 |  |
| No | 131 | 53 |  |
| Chemotherapy regimens |  |  | 0.2597 |
| 1MTX, DDP and ADM | 100 | 39 |  |
| 2MTX, IFO,DDP and ADM | 27 | 14 |  |
| 3MTX,IFO, DDP and ADM | 10 | 1 |  |

Note: Individual clinical factors were analyzed for significant differences using a chi-square test. P < 0.05 indicates a significant difference.

**IV. Supplementary Figure S3** Heatmap for radiomic features.


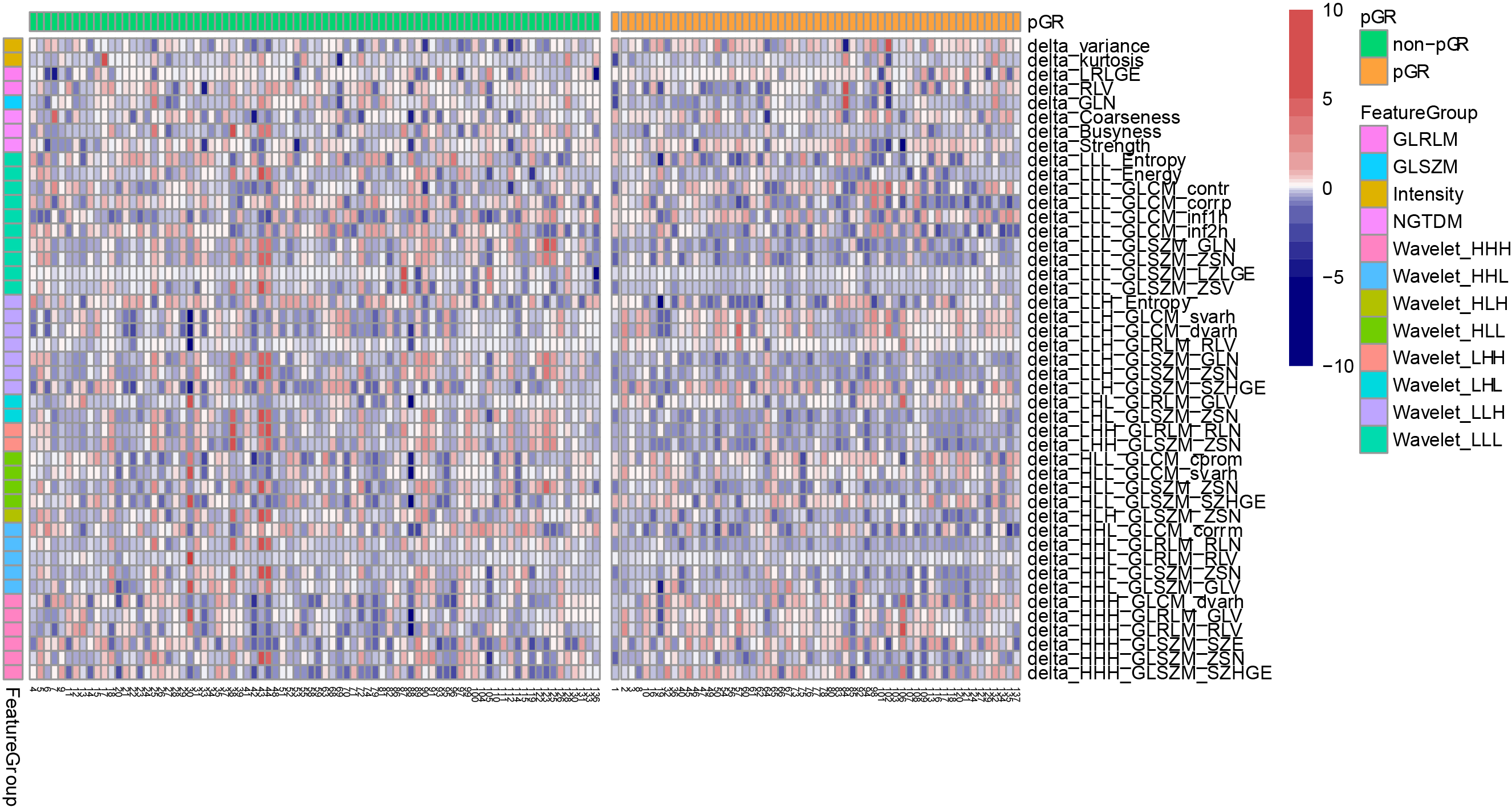


**Figure S3.** Heatmap for instructive radiomic features in the training set. The x-axis indicates different patients. The y-axis indicates different radiomics features. The color in the box shows the expression level of radiomic features.

**V. Table S2:** Interclass correlation coefficient (ICC) values of selected delta-radiomic features in the intra-observer and inter-observer reproducibility test.

| Feature Name | ICC in  Intra-observer Test | ICC in  Inter-observer Test |
| --- | --- | --- |
| △variance | 0.998 | 0.997 |
| △LLL_GLCM_corrp | 0.990 | 0.983 |
| △LLH_Entropy | 0.984 | 0.934 |
| △LLH_GLSZM_GLN | 0.996 | 0.992 |
| △LHH_GLSZM_ZSN | 0.945 | 0.962 |
| △HHL_GLCM_corrm | 0.801 | 0.806 |
| △HHH_GLSZM_SZE | 0.926 | 0.926 |
| △HHH_GLSZM_SZHGE | 0.891 | 0.926 |

**VI. Supplementary Figure S4** The predictive performance of the radiomic signatures from four kinds of data.

**
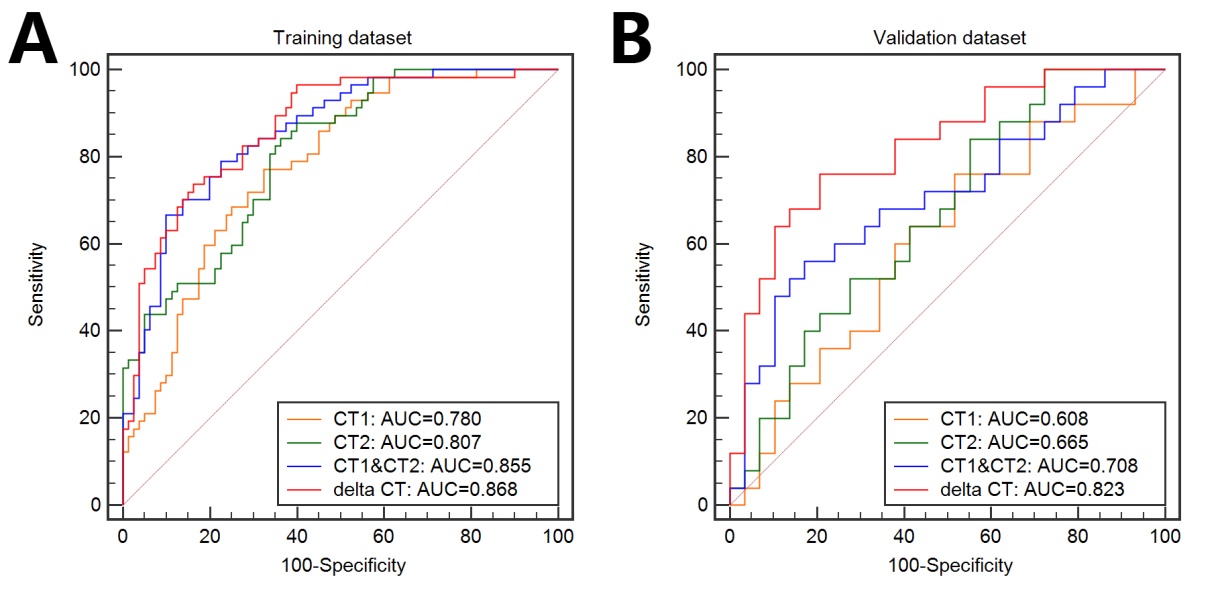
**

**Supplementary Figure S4.** The predictive performance of the radiomic signature from four kinds of data for each patient in training (A) and validation (B) sets (AUC, area under curve).
